# Dynamic Viral Glycoprotein Machines: Approaches for Probing Transient States That Drive Membrane Fusion

**DOI:** 10.3390/v8010015

**Published:** 2016-01-11

**Authors:** Natalie K. Garcia, Kelly K. Lee

**Affiliations:** Department of Medicinal Chemistry, University of Washington, Seattle, WA 98195, USA; nkgarcia@uw.edu

**Keywords:** viral membrane fusion glycoprotein, structural mass spectrometry, electron microscopy, small-angle X-ray scattering, hydrogen-deuterium

## Abstract

The fusion glycoproteins that decorate the surface of enveloped viruses undergo dramatic conformational changes in the course of engaging with target cells through receptor interactions and during cell entry. These refolding events ultimately drive the fusion of viral and cellular membranes leading to delivery of the genetic cargo. While well-established methods for structure determination such as X-ray crystallography have provided detailed structures of fusion proteins in the pre- and post-fusion fusion states, to understand mechanistically how these fusion glycoproteins perform their structural calisthenics and drive membrane fusion requires new analytical approaches that enable dynamic intermediate states to be probed. Methods including structural mass spectrometry, small-angle X-ray scattering, and electron microscopy have begun to provide new insight into pathways of conformational change and fusion protein function. In combination, the approaches provide a significantly richer portrait of viral fusion glycoprotein structural variation and fusion activation as well as inhibition by neutralizing agents. Here recent studies that highlight the utility of these complementary approaches will be reviewed with a focus on the well-characterized influenza virus hemagglutinin fusion glycoprotein system.

## 1. Introduction

Enveloped viruses bear a host-derived lipid membrane that encapsulates and protects the viral genetic material. To infect new cells and deliver the viral genome into the host cell cytoplasm, the virus must open a conduit across the membrane barrier by merging its lipid envelope with a host cell membrane. Fusion glycoproteins (abbreviated gp or GP depending on virus type) anchored to the virus membrane and displayed on the virus surface mediate this essential process. To understand the function of these cell entry machines requires detailed structural characterization and elucidation of the conformational transitions and intermediate states that are populated during the fusion process. In addition, many of the viruses, particularly those with RNA genomes, exhibit significant variation due to mutational drift and selection. The differences in amino acid sequence impact glycoprotein structure, antigenicity and fusion protein function during cell entry. Yet our understanding of the link between sequence, structural variation, phenotype and function are still in their infancy.

Viral fusion glycoproteins are often challenging targets for high-resolution structural characterization due to their decoration with flexible and heterogeneous glycans, intrinsic structural dynamics, and instability of the native, metastable pre-fusion states. In recent years, a diverse array of biophysical methods have been applied that: (i) provide complementary insights into the nature of conformational changes during fusion activation and neutralizing antibody engagement [1,2,3,4,5,6,7,8,9,10,11,12,13]; (ii) reveal the flickering, transient conformational sampling exhibited by glycoproteins on the surface of virions [12,14]; and (iii) illuminate variations in structural dynamics that impact the antigenicity and receptor reactivity of viral glycoprotein components [15,16,17]. In this review, we will describe a set of biophysical methods that have recently provided new perspectives on viral fusion protein structure and function, and present the influenza hemagglutinin (HA) system as a prime example of how the various methods can combine to give a more complete understanding of this dynamic fusion machine. We also highlight developing areas that may benefit from application of these new approaches.

Influenza HA is the best characterized type-I fusion protein, with high-resolution structures of pre- and post-fusion conformations [18,19], a multitude of neutralizing antibody fragment antigen binding (Fab) domains that have been characterized in complex with the glycoprotein antigen [20,21,22,23,24,25,26,27,28,29], a detailed 3-dimensional understanding of virus ultrastructure [30,31,32,33,34], and decades of biophysical characterization [1,11,13,35,36,37,38,39,40,41,42,43,44,45,46,47]. Despite this wealth of information, until recently we have lacked structural information describing the sequence of changes exhibited by hemagglutinin during fusion, and even basic information for the nature of target and virus membrane deformations that hemagglutinin induces in order to drive membrane fusion is unclear.

**Figure 1:**
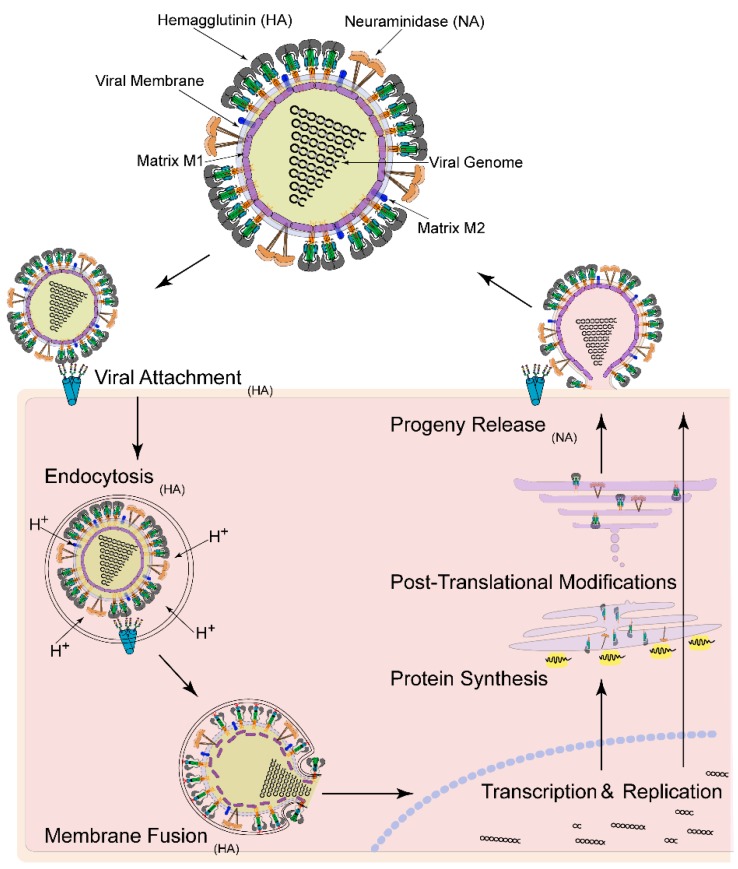
Influenza virus structure and infectious cycle. Viral attachment is mediated by Hemagglutinin (HA), which bind through multi-valent, high avidity interactions to host cell surface glycans, triggering endocytosis of the virus particle. Following acidification of the endosomal lumen, M1 dissociates from the inner surface of the viral membrane, and HA undergoes large structural changes to drive membrane fusion ultimately releasing the nucleoprotein-coated genome segments into the cytosol, which is transported to the nucleus for genome transcription and replication. Viral proteins are synthesized and post-translationally modified via the endoplasmic reticulum and trans-golgi network. Progeny viruses are assembled at the cell surface, where Neuraminidase (NA) cleaves neighboring sialic acid for release of newly formed virus into the extracellular milieu.

Like other type-I fusion proteins, HA is a homo-trimer of hetero-dimeric protomers [18]. One subunit in the protomer, HA1, is responsible for receptor binding, and a second, HA2, is the primary fusion machinery. In analogous fusion systems such as the human immunodeficiency virus (HIV) envelope glycoprotein (Env), receptor binding induces major conformation changes in the receptor binding subunit [48,49,50,51,52,53], which are transduced to the fusion subunit leading to its priming and activation [2]. HA attachment to extracellular sialic acids on glycoproteins and glycolipids on the cell surface initiates the influenza virus infection cycle (Figure 1) [54,55,56,57]; however, no evidence has been found to indicate that sialic acid receptor binding induces a structural change or activation of the HA fusion machinery. The low, ~mM affinity of sialic acid for the receptor binding site suggests that high avidity binding of three sialic acid binding sites per trimer, multiplied over several trimeric spikes on a virus-host membrane contact surface is needed to adsorb the virus to the cell surface [58,59,60]. 300–500 of the trimeric HA spikes are present on typical influenza virus particles, in ~5-fold copy number excess over the other surface protein, neuraminidase, which has an essential sialidase activity needed to release newly budded virions from the cell surface [30,61,62]. Multivalent sialic acid receptor engagement through HA appears to trigger endocytosis of the virus, rather than entry occurring at pre-existing endocytosis hotspots [46,47].

Influenza HA activation is triggered by exposure to the acidic pH within maturing endosomes (pH~6.0–5.0), resulting in the complete structural rearrangement of the fusion protein [18,19,46,57,63,64]. Early differential scanning calorimetry experiments indicated that HA is kinetically trapped in a metastable conformation, which upon conversion to the post-fusion state, releases conformational energy to help drive membrane apposition and fusion [35,36,37,38,39,57,64,65,66]. Carr and Kim first described the mechanism as a “spring-loaded” change [35] in which a loop in pre-fusion HA irreversibly becomes helical at low pH in order to extend the central helical bundle and relocate the fusion peptide at the N-terminus of HA2 from close to the base of the trimer ~100 Å towards the viral membrane. The structure of a post-fusion HA soluble fragment (TBHA2) where BHA was treated with low pH and proteolyzed to remove HA1 and the fusion peptide also revealed that HA2 refolding involved a helical break that permits the C-terminal portion of the subunit to repack along the newly extended core [19]. In a related study of HIV gp41 by Weisenhorn *et al.* [67], it was proposed that this refolding of the C-terminal segment of the fusion subunit along the central helical bundle drives membrane fusion. Indeed in the post-fusion state, the N-terminal fusion peptide and C-terminal membrane anchor region are colocalized, consistent with their role in juxtaposing the two membranes to induce their fusion. When HA2 was expressed recombinantly in *E. coli* the fold observed in the post-fusion TBHA2 structure was found to be the low energy conformation for the HA2 polypeptide sequence [68]. Subsequent analysis suggests that prior to the adoption of the ultimate post-fusion state, reversible stages of conformational change exist and these appear to play important roles in initiating engagement of the target membranes [32,38,40,41,64,69,70,71].

In addition to influenza HA, high-resolution structures have been determined for other type-I fusion glycoproteins in their pre-fusion states including paramyxovirus F proteins [72,73,74], major portions of the Ebola glycoprotein (GP) [75,76], and the HIV Env glycoprotein in an engineered, mutation stabilized form, allowing identification of receptor binding sites and organization of the receptor binding and fusion subunit [7,8,14,77]. This information has proved exceptionally informative for understanding the viral spike structures and disposition of epitopes for neutralizing antibodies, which most frequently target the pre-fusion conformation of the glycoprotein antigens on the virus surface.

Structures of fragments of the proteins in the low-energy, post-fusion conformations have also been reported. The post-fusion “hairpin” structures of type-I fusion proteins such as HIV Env gp41 and Ebola GP2 revealed commonality of presumed fusion mechanisms, highlighting the end-stage colocalization of N and C-terminal, membrane-active subdomains in the post-fusion conformation [19,67,68,78,79,80,81]. Metastability of the pre-fusion conformation and the low energy, ground state character of the post-fusion conformation are believed to be common traits for type-I fusion proteins. What remains for all of these systems is to understand the pathways of conformational change that link the beginning and end states, and that in fact actively manipulate membranes and drive the fusion reactions to completion. Such a challenging task is becoming tractable through the development of biophysical methods that enable proteins in dynamic, transient states to be analyzed under a broad range of solution conditions.

## 2. Solution-Based Biophysical Approaches

**Figure 2:**
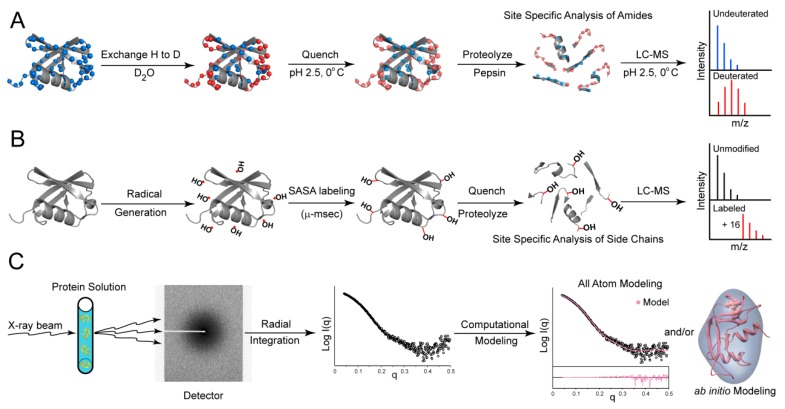
Solution Based Approaches for Protein Characterization (**A**) Hydrogen-Deuterium Exchange Mass Spectrometry (HDX-MS). Exchange of backbone amide protons for deuterons is initiation by incubating a protein in deuterated buffer for various amounts of time, reaction is quenched at pH 2.5 and on ice, samples are denatured and digested, and mass shifts are analyzed by mass spectrometry; (**B**) Oxidative Labeling MS. Biological macromolecules in solution are exposed to hydroxyl radicals for various periods of time, which covalently modify solvent accessible surface area (SASA) via amino acid side chains. Radicals can be rapidly generated by radiolysis of water or photolysis of hydrogen peroxide, or slowly generated using Fenton’s reagents. The reaction is quenched using radical scavengers and the samples are proteolyzed and analyzed by mass spectrometry; (**C**) Small Angle X-ray Scattering (SAXS). Biological macromolecules in solution are irradiated with a monochromatic X-ray beam, which generate a characteristic scattering pattern. Computational methods such as all-atom modeling can be used to compare high-resolution structures to the solution-phase data, or *ab initio* shape reconstruction can be used to generate low-resolution morphological information.

Solution-phase protein labeling experiments in conjunction with mass spectrometry (MS) is a growing field in structural biology (Figure 2A,B), providing sequence-specific information about native protein conformational dynamics and structural organization. These approaches, including hydrogen/deuterium-exchange mass spectrometry (HDX-MS) and oxidative radical footprinting followed by MS analysis, can be applied to a broad range of proteins, glycoproteins, membrane-bound proteins and even proteins in the context of whole virus particles [1,82,83,84,85,86,87]. Protein size is less of a limiting factor than for example with nuclear magnetic resonance (NMR) spectroscopy, which also can be used to probe structural dynamics of smaller macromolecules but is less suitable for analysis of objects in the size range of viral glycoproteins. With the advent of more advanced mass spectrometers such as those employing ion mobility separation for enhanced identification of peptide fragments, it is becoming possible to analyze even more complex targets [88]. The mass spectrometry-based methods are particularly powerful when applied to compare differences resulting from changes in solution conditions, or between two related variants such as mutant and wild-type proteins, or proteins in ligand-bound and free states, where for example one can map antibody epitopes within an antibody-antigen complexes [89,90,91,92].

## 3. Hydrogen-Deuterium Exchange Mass Spectrometry (HDX-MS)

HDX-MS (Figure 2A) probes local structural dynamics in native proteins and glycoproteins yielding a “fingerprint” of structural dynamics with sensitivity necessary to identify isolate-specific differences in structure and the ability to identify ligand-induced structural changes [15,16,17,93]. By itself this approach does not provide 3-dimensional structural information; rather one obtains a measurement of structural dynamics or flexibility of segments of the protein as they exist under native conditions in the folded protein. In combination with other available structural information, HDX-MS can be a powerful method for mapping conformational changes and characterizing structures that may be too dynamic and flexible to characterize by crystallography or electron microscopy (EM).

HDX-MS measures the rate of deuterium incorporation at the backbone amides, which is dependent upon fluctuations in local structural order. The apparent deuterium exchange rates of each amide in the protein polypeptide backbone is predominately influenced by hydrogen bonding, such as in secondary structure formation, while solvent occlusion plays less dominate role [89,93]. The protein is incubated in D_2_O-based buffer under native conditions for a range of times, after which, the solution is acidified to pH 2.5 to slow deuterium exchange at the amides. Structural analysis of the protein requires proteolytic digestion of the partially deuterated protein under quench conditions using acid-active proteases such as pepsin. The peptic fragments are resolved chromatographically and analyzed by mass spectrometry. The extent of deuterium up-take for each peptide is interpreted by the mass shift as a function of deuteration time.

The reliance upon the acid-active protease, pepsin, imposes one major limitation in HDX-MS analysis in the form of non-uniform sequence resolution. Pepsin reproducibly cleaves the polypeptide into a range of segments of varying length [94]. As a result, one obtains peptides that range from a few to tens of residues in length. Addition of methods such as electron transfer dissociation (ETD) for the further fragmentation of peptides in the mass spectrometer can in some cases increase resolution down to individual residues [95]. Thus far, ETD has only been applied for a few studies, but with increasing availability of appropriate instrumentation and software development it is becoming of greater utility for HDX-MS [96,97]. The use of acid-active protease that cleave with different specificities to pepsin may offer a way of providing complementary peptide coverage and generating an overlapping mosaic of exchange profiles based on the two separate or combined proteolytic activities [98,99].

In the analysis of glycoproteins, resistance to proteolysis and poor signal for glycopeptides complicates analysis [100,101]. Hyperglycosylated regions within a protein typically generate large peptic fragments that may contain multiple glycans per peptide. In some cases the highly glycosylated peptides bind poorly to the reverse-phase columns implemented during liquid chromatography. Microheterogeneity of glycoforms at each *N* or *O*-linked glycosylation site results in the signal for a glycosylated peptide to be distributed among several glycoform variants, and in the case of poorly ionizable peptides, can significantly hamper detection.

While the vast majority of structural information for influenza HA as well as other viral glycoproteins has come from high-resolution structural analysis of soluble constructs produced either recombinantly or by proteolytically cleaving the proteins from intact virus, it is important to confirm that the soluble form of the protein retains the native structure found on the intact virion. For influenza HA this has been now demonstrated at low-resolution using cryo-electron tomography [30,31,34] as well as in greater, sequence-specific detail by HDX-MS in which soluble, bromelain-released HA (BHA) from A/Aichi/68/H3N2 was shown to exhibit a nearly superimposable HDX profile as HA on intact virus particles at neutral pH [1].

The ability to vary solution conditions and analyze structure by HDX-MS enabled mechanisms of influenza HA activation to be investigated by comparing the structure of HA under pre-fusion and post-fusion conditions and under pH conditions approaching the threshold for activation [1]. This has helped to provide a more complete understanding of the post-fusion organization of the full HA ectodomain. High-resolution structural characterization of post-fusion HA is limited to the HA2 ectodomain and does not provide information about the post-fusion HA1 conformation. HA1 monomers proteolyzed from acid-treated viral HA, complexed with an antibody Fab domain and crystallized at pH 6.0 indicated that the overall morphology of HA1 remains similar to that seen in the pre-fusion trimer [23]. HDX-MS analysis of HA1 in the context of the BHA trimer ectodomain, however, exhibited much greater levels of structural dynamics than observed in the pre-fusion state suggesting the HA1 receptor binding subunit loses much of its integrity following conversion to the post-fusion state.

**Figure 3:**
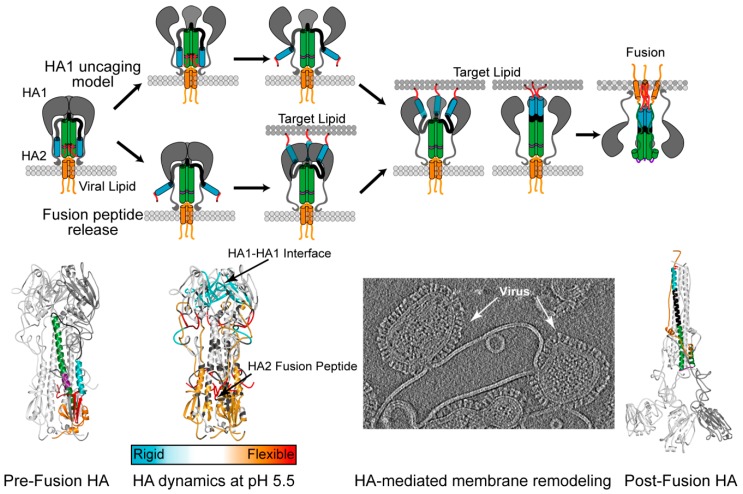
Influenza Hemagglutinin pH-Activation Models. Pre-fusion HA is composed of the two subunits, HA1 (**grey**) and HA2 fusion domain with the fusion peptide and proximal regions (**red**), the short helix (**blue**), the B-loop (**black**), the long helix (**green and purple**), and the C-terminal regions (**orange**), colored identically on 3HMG crystal structure below. The pH-dependent membrane fusion is hypothesized to commence either by the uncaging of HA1 (**top pathway**) from the HA2 fusion machinery, or by the release of the fusion peptide from the core of the trimer (**bottom pathway**). Once the fusion peptide is released, it binds the target membrane and the subsequent refolding of the B-loop and long helix (**purple**), drives the fusion of the viral membrane with the target membrane. Membrane fusion ends in the post-fusion conformation depicted in the bottom panel where HA1 lobes are modeled onto the 1QU1 crystal structure. Recent solution-based techniques such as HDX-MS and cryo-electron tomography have provided evidence for the fusion peptide release model for H3 HA isolate fusion activation. The HDX dynamic changes are illustrated as regions more flexible at low pH (**red**) colors and more ordered regions at low pH (**blue**). In the cryo-electron tomogram, acid-activated influenza virus is shown at early stages of membrane fusion with a synthetic liposome.

HDX-MS analysis of BHA also demonstrated that at pH values approaching fusion activation for this isolate (pH 5.5), major increases in local conformational flexibility were observed at the fusion peptide and HA1 hinge proximal to the HA1-HA2 interface (Figure 3) [1]. The use of an internal standard peptide containing a single exchangeable site along with incubation time adjustments was necessary to account for the pH-dependence of the intrinsic exchange rates during labeling [1,102]. Previous structures obtained from HA mutants crystallized at conditions approaching fusion, suggested that rotations within the B-loop and adjacent HA1 residues may, in part, initiate HA activation [69,103]. Some of the local structural changes detected by HDX-MS echoed these results, however, the solution-phase analysis of the H3 Aichi/68 BHA demonstrated that the fusion peptide and associated structural regions become highly dynamic as the pH of fusion is approached, which was not observed by crystallography [69,103]. Furthermore, the HDX-MS data revealed moderate stabilization of the HA1-HA1 trimeric interface under fusion-activation conditions, in apparent contradiction of HA1 domain dissociation or “uncaging” of the HA2 fusion subunit, which is often implicitly assumed to initiate the fusion process (Figure 3). Instead, the recent data suggests that HA activation is initiated by fusion peptide release and reorganization, which does not require a large-scale opening of the trimeric spike. This pathway would appear to allow fusion peptides to bind target membranes prior to unleashing the full HA2 “spring-loaded” transition.

Recent cryo-electron tomograms of influenza virus acidified to pH 4.9 and frozen after 5 min at room temperature provided a low-resolution glimpse of an HA intermediate that is consistent with HDX-MS data, displaying a narrowing of the HA2 stalk region while density for the globular head remained intact [32]. As with all low-resolution methods, validation by orthogonal methods and testing for consistency against available biochemical data is desirable. Investigations characterizing HA activation intermediates by White and Wilson in 1987 used antibodies that recognize specific conformational epitopes on HA [104]. The experiments showed that the fusion peptide release from the fusion peptide pocket precedes exposure of epitopes at the HA1 globular head domain interfaces that are occluded in the pre-fusion structure. In a follow-up study by Kemble *et al.* [105], it was reported that fusion peptide exposure was concurrent with minor changes in the membrane distal apex of the trimer that occurred prior to HA1 dissociation (Figure 3). This antibody mapping data is thus consistent with the model suggested by HDX-MS for changes in HA during acid-induced fusion activation at least for the A/Aichi/68 H3 HA that has been most intensively examined. It remains to be determined whether HA from other subtypes exhibits similar changes upon activation. We note that crystallography was performed with two “group 1” HA trimers, from H2 and H5 isolates, while the HDX-MS analysis examined a “group 2” HA from an H3 virus. Given the different propensities to inactivation that have been reported for different viral isolates [13,41,42,71], it may be the case that the HA stability, fusion activation and transience of intermediate conformations vary between isolates. Further studies assessing the isolate-specific dynamics during fusion activation are needed to truly answer these remaining questions.

Indeed in the case of highly variable viruses such as HIV and influenza, it is desirable to understand the structural basis that governs the functional and antigenic properties for each isolates, in hopes to find a common therapeutic target [13,42,106,107]. The ability to analyze protein structure under native solution conditions for glycoproteins by HDX-MS has made it possible to perform comparisons of glycoprotein constructs from highly divergent viral isolates [2,15]. The structural differences that are evident when glycoproteins such as HA and HIV Env are studied under native solution conditions are often suppressed by removal of variable elements, partial deglycosylation, complex formation with stabilizing ligands such as Fabs and the constraints of crystallization itself [15,53,108].

HDX-MS has also provided significant new information about the initial stages of HIV Env activation upon CD4 receptor binding [2,15,52,53], which had previously been inferred at low-resolution from EM studies [4,5,6,109] or crystal structures of truncated subunit constructs [49,50]. HDX-MS was used to compare native-like Env trimers (SOSIP.664 constructs [10]) in an unliganded and in CD4-bound forms (using a soluble, 2-domain form of CD4) [2]. Illustrating the specificity of structural perturbations that can be detected by this method, two distinct allosteric networks were found to be engaged by CD4 binding: one leading to opening of the trimer apex and coreceptor binding site exposure, and a second involving priming of the gp41 fusion subunit. HDX-MS has also distinguished the distinct binding modes for two CD4 binding site-targeted broadly neutralizing antibodies (bNAbs), VRC01 and b12. These comparisons revealed the trimer stabilizing effect in the case of VRC01 and the apparent destabilizing effects of b12 [3]. The HDX-MS observations of the soluble trimeric Env constructs in complex with highly potent bNAb and CD4 are consistent with recent studies using single-molecule Förster resonance energy transfer (sm-FRET) to monitor the dynamic conformational sampling of HIV Env on the surface of virus particles in complex with CD4 and a panel of bNAbs including VRC01 [12].

Analysis of conformational dynamics and structural order by HDX-MS of viral glycoproteins from other enveloped viruses has been reported recently as well. HDX-MS was used to examine the impact of proteolytic processing of the Ebola virus GP and dynamic changes within antigenically distinct GPs [16,17]. Identification of flexible residues within the hypervariable regions of a deglycosylated ectodomain construct of the E2 glycoprotein from hepatitis C virus (HCV) was made possible with the aid of HDX-MS [110]. This highlights another useful application of HDX-MS in identification of highly flexible, dynamic regions that can be truncated to facilitate crystallization. The synergy between HDX-MS and electron microscopy was also useful for identifying the architecture of the human cytomegalovirus (HCMV) glycoprotein entry complexes and for identifying the discontinuous conformational epitope of a neutralizing antibody [111]. Investigations of temperature-dependent changes in global and local structure as probed by cryo-EM and HDX-MS in the context of an intact dengue virus particles have also been reported [84]. These varied studies highlight the versatility of HDX-MS for structural and functional analysis of diverse viral glycoproteins under a broad spectrum of solution conditions.

Lastly, HDX-MS has utility in validating whether proteins are natively folded. This is an important consideration in the development of subunit-based immunogens for viral vaccines. The ability for a protein-subunit vaccine construct to elicit an antibody-immune response is likely contingent upon having properly folded immunogens that present authentic epitopes to the immune system. HDX-MS can provide rigorous assessment of whether proteins are natively folded and in the expected conformation and organization [112,113]. Such approaches have been gaining utility in the analysis of protein biologics with respects to storage and formulation conditions, which may become a gold-standard required by the Food and Drug Administration [101,114,115,116].

## 4. Oxidative Labeling With Mass Spectrometry

Complementary to HDX-MS, which is highly influenced by secondary structure, oxidative labeling analyzed by mass spectrometry (Figure 2B) utilizes hydroxyl radicals generated rapidly by either synchrotron X-ray radiolysis of water or ultraviolet laser photolysis of hydrogen peroxide to covalently modify solvent accessible amino acid side chains in proteins and in complexes [82,90,117,118,119]. By probing side-chain reactivity to radicals in bulk solvent, oxidative radical labeling allows solvent-accessible protein surfaces to be mapped [85,87,118,119,120,121]. In addition, the versatility of the both methods to be performed under a broad range of solution conditions enables the analysis of large dynamic protein systems that require atypical environments (e.g., detergents, low pH) [82,86].

The oxidative labeling approach has revealed conformational changes in viral antigens such as in a domain from the HIV gp120 receptor binding subunit [122]. It has been used to map interaction interfaces with neutralizing antibodies, as well as to probe the structural changes in trimeric HIV Env in response to CD4 binding [2,123]. More recently, a study combining temperature-activation and oxidative labeling of the fusion protein from parainfluenza virus 5, identified differences in solvent accessibility and structural reorganization between pre-fusion and post-fusion conformations [124].

A limitation of the oxidative labeling approach is that different amino acid side chains have different intrinsic reactivities with radicals. The residues one can monitor may be unevenly and infrequently distributed throughout the protein sequence and structure, giving rise to relatively sparse sampling of solvent accessibility throughout the folded protein. The ability for other solution components to react and scavenge the radicals should also be considered during experimentation [90,125,126]. Proteins are also susceptible to oxidative damage, and rapid generation and labeling times are necessary to prevent denaturation during labeling, which may convolute interpretation [87,121].

## 5. Small Angle X-Ray Scattering (SAXS)

SAXS (Figure 2C) is useful technique that involves the measurement of elastic X-ray scattering from macromolecules in solution. The SAXS pattern one obtains is directly related to the 3-dimensional organization of atoms in the scattering object, hence one can extract structural information from the measured SAXS data [127,128]. In the most elementary application, SAXS can be used to determine accurate protein and glycoprotein molecular weights [15,52,113,129,130,131,132,133]. Radii of gyration and maximum point-to-point dimensions of the scattering object can also be readily obtained. With *ab initio* shape reconstruction programs, the scattering pattern can yield low-resolution morphologies of proteins and protein complexes in solution (Figure 2C) [128,134,135,136].

A particularly powerful approach has come into use more recently in which one builds upon sometimes fragmentary high-resolution structural information by modeling in missing features and iteratively changing and selecting models that produce optimal agreement with experimentally measured SAXS data [137,138,139,140,141]. It is important to note that the solution scattering profile results from the entirety of scattering correlations of atoms in the object of interest, hence it is essential to model in not just missing protein loops but to include glycans and other post-translational modifications as well [132,133]. With the experimental SAXS pattern as a constraint, in simpler cases, it is possible to identify ensembles of models with glycan orientations that show a better agreement with the measured data, allowing the spatial occupancy of the glycans to be estimated [132]. In *ab initio* modeling, due to averaging effects and limitations on resolution, glycans tend to not be well resolved [52,132].

When applied to examine structural integrity and changes in conformation during acid-induced activation of bromelain-released HA, SAXS demonstrated that at pH 5.25, the spike ectodomain largely retained its pre-fusion-like organization, *i.e.*, without HA1 domain dissociation and HA2 springing to the helical bundle post-fusion state, though subtle structural changes that were consistent with the more detailed HDX-MS results were observed [1]. SAXS has been applied to analyze glycosylated HIV Env gp120 subunits, which at ~110 kDa including 50% glycans by mass are generally too small for detailed EM analysis and too large for NMR [15,52,142]. SAXS revealed the orientation of the large V1/V2 hypervariable loops relative to the gp120 core and showed that V1/V2 shift in position following CD4 binding [52]. SAXS also demonstrated that while they may exhibit significant differences in local structural order, divergent gp120s retain similar global organizations [15]. SAXS thus fills an important niche in providing structural characterization, albeit at low-resolution, of even relatively small glycoprotein constructs, and is considerably less limited on the other end of the spectrum for analysis of large proteins and complexes including even whole icosahedral capsids [143,144,145,146,147,148,149].

SAXS has also been used to map the position of a neutralizing antibody Fabs bound to a trimeric form of HIV Env, showing good agreement with a low-resolution negative stain EM reconstruction [150]. The implementation of SAXS was also useful for identifying different binding modes of a cross-reactive neutralizing human antibody to the heavily glycosylated filovirus GP glycoprotein from Marburg and Ebola, which were refractory to crystallography [149]. In examination of receptor binding interactions with HCV E2 glycosylated ectodomain and core constructs, SAXS proved useful in interpretation of possible binding models [110].

## 6. Electron Microscopy (EM)

EM is experiencing major advances in both attainable resolution and an increasingly powerful ability to grapple with heterogeneous, conformationally variable samples over a range of sizes from ~200 kDa to megadalton structures [151,152]. Because the specimens do not require crystallization, they can be examined under a range of solution conditions and complexed with a range of ligands. By single-particle analysis, starting with relatively pure samples, a 3-dimensional reconstruction of viral glycoproteins alone or in complex with antibody Fab fragments can be obtained [153]. By electron tomography (ET), complex, asymmetric and heterogeneous specimens can be imaged to characterize virus ultrastructure [154,155,156]; this is useful for example in imaging glycoprotein distribution, orientation and interaction with other ligands or receptors.

Much like an X-ray CAT (computer assisted tomography) scans, ET enables 3D reconstructions to be built from 2D projections gathered at different specimen tilt angles. Cryo-ET has also been used to probe the structure of diverse enveloped viruses [30,34,157,158,159,160,161,162,163,164,165,166,167]. The method is also a powerful means for imaging biological processes such as virus undergoing membrane fusion with vesicles [31,168,169], virus entry into intact cells grown on EM grids [170], virus budding from cells [171,172,173], and virus maturation [174,175,176,177], to name just a few fundamental aspects of enveloped virus biology. With cryo-ET, resolution is typically limited to ~10–20 Å, and due to the incomplete sampling of angular orientations (mechanical constraints limit tilt angles to ~±70°), information for the top and bottom of objects tend to be poorly represented in the final reconstructions. However, in many cases, density in reconstructed electron tomograms corresponding to surface glycoproteins can be clearly resolved and is often found to be in excellent agreement with available high-resolution crystal structures [4,30,31,162,178].

In imaging influenza HA under fusogenic conditions, cryo-ET has proven to be a uniquely well-suited approach that enables virus-liposome complexes to be imaged with resolution of membrane leaflets, coordinated HA spikes and other viral components such as the M1 matrix layer that plays a critical role in virion assembly as well as during fusion [31,32,33]. Intriguingly, at many of the sites of nascent pore formation, when the target membrane is being drawn as a dimple towards the virus envelope by a localized cluster of HA, distinct “V” or “Y” shaped densities are observed coordinating the dimple [31]. This suggests an intermediate state of HA undergoing the refolding process with the central HA2 helical bundle being extended but the C-terminal “leash” parts of the subunit that are anchored to the viral membrane only partially docked into the grooves of the bundle [31,179]. Observation of such intermediate structures is likely only feasible when full-length HA natively presented on a membrane is grappling with the target membrane.

Sub-tomogram averaging can be applied to enhance definition of glycoprotein density. This method involves “boxing” out sub-volumes from larger 3D reconstructed fields of view and averaging the individual boxed structures to increase signal-to-noise of common density features while suppressing density for conformationally variable elements [180]. Cryo-ET with sub-tomogram averaging has addressed important controversies in HIV Env structure [4,181,182,183,184,185], and been used to map the binding of neutralizing antibodies to viral glycoproteins on intact virions [4,5,186] for example. Using cryo-ET to study filovirus glycoprotein ultrastucture, the large, heavily glycosylated mucin domain, which typically is truncated from constructs used in crystallization, could be localized [187], and glycoprotein organization on the surface of virus particles was readily apparent from reconstructed density maps [162]. Recent reconstructions of Gag structures on the inside of retrovirus particles have yielded striking clarity at ~8 Å resolution, revealing for example helical sub-structures in portions of the Gag lattices [188,189]. Gag adopts a repeated lattice organization inside of particles, which helps constrain the individual protein copies while facilitating averaging [180]. While it may be challenging to achieve such resolutions for the more dynamic, heterogeneous surface glycoproteins, the Gag studies highlight what is achievable with sub-tomogram averaging under optimal circumstances with rigorous and thorough processing and analysis.

Single particle analysis, that is typically performed on purified proteins and complexes is now enabling structural biologists to achieve near-atomic resolution of protein assemblies without requiring crystallization of the glycoprotein of interest [7,77,190,191]. This approach does not require proteins to be deglycosylated or to have loops truncated, which often are necessary to produce constructs amenable to crystallization. One images fields of proteins flash-frozen in vitreous ice, and reconstructs a 3-dimensional image based upon the individual projection images of the individual particles. Ideally the particles are randomly oriented, providing a thorough sampling of views of the 3-dimensional proteins. In the reconstruction process, it is necessary to determine how the different views relate to each other and to the 3-dimensional model. Fabs bound to proteins in the size range of common viral antigens (~200–400 kDa) can provide prominent features that aid in the determination of particle orientations from relatively noisy micrographs [192]. Remarkably, by single-particle analysis it is becoming possible to resolve significant portions of glycan chain density with the use of state-of-the-art imaging and analysis approaches [7,191].

In some cases, samples may not be amenable to cryo-EM preparation, or one may not require the detailed insights offered by high-resolution structure determination. In these cases, negative-stain EM with single particle analysis can provide valuable information. Here, samples are adsorbed to carbon-coated EM grids and a thin layer of a heavy metal stain coats the specimens. The samples are no longer under native conditions as in cryo-EM, but staining often helps to fix the specimens, providing a high-contrast cast of their structure. When imaging flexible proteins using negative stain EM, adsorption to the carbon substrate, sample dehydration, and stain-protein interactions can potentially deform the objects of interest. Thus, it is useful to validate the negative stain EM models against all available structural information gathered by other methods such as X-ray crystallography.

Nonetheless, negative stain EM with single particle reconstruction has been useful for example in identifying the organization of Ebola GP-antibody complexes from a panel of antibodies comprising the ZMapp antibody cocktail, which is being used to treat the 2014 West Africa outbreak [193]. Epitope surfaces could be localized and general Fab orientations and approach angles were clearly evident in the low-resolution structures. Likewise in numerous studies of influenza HA, HCV, Marburg, and Ebola glycoproteins as well as soluble, engineered forms of HIV Env trimers, negative-stain EM has provided valuable models that reveal the epitope surfaces recognized by antibody Fabs as well as the relative orientation of Fab and antigen in the complex [10,149,150,153,193,194,195,196,197,198,199,200].

It is useful to hold some notable caveats in mind when using electron microscopy reconstructions for interpretation of biological data: the beautiful 3-dimensional models are constructed from images of a subset of the total number of particles in a given population, sometimes a small minority of the total particles. Species bias may be present in terms of the particle types or species that favorably position themselves in the EM grid holes or that adsorb to carbon substrates in the case of negative stain EM imaging. In the process of optimizing single particle EM reconstructions, selection of particles that are more similar to each other and improve the resulting reconstruction also winnows the population of particles that are used in composing the final model. Here it is useful to avoid assuming that the end model one obtains necessarily is representative of the population that one started with in a specimen. As classification methods improve, and sense can be made of heterogeneous samples, the strength of cryo-EM in imaging individual particles opens new windows into studying conformational variability and structural dynamics [152]. The reader is also referred to recent discussions that describe the pitfalls and challenges in EM image analysis both in single-particle cryo-EM analysis [201,202,203] and in cryo-electron tomography [4,181,182,183,184,185].

Cryo-EM has been useful for resolving the internal and external architecture of complex, enveloped viruses, revealing how the many components—glycoproteins, matrix proteins, ribonucleoprotein complexes, and membranes—are integrated into an infectious particle [30,31,32,33,204,205,206,207,208]. For some icosahedrally symmetrical particles, such as many of the flaviviruses, cryo-EM is providing resolution to rival crystallography. A 4.4 Å resolution structure of the Venezuela equine encephalitis virus was able to identify novel structural features not observed by crystallography and validate *de novo* models of the E1, E2 fusion protein complex in an infectious particle [209]. Different serotypes of dengue viruses have also been solved to near-atomic resolution, which were capable of distinguishing isolate specific differences, and identifying structural modifications necessary for viral maturation and pH-dependent fusion [205,206,208].

## 7. Conclusions

Taken together, these reports highlight the diverse application of solution-phase techniques for structural analysis and structure determination, which in combination with known high-resolution data can be used to investigate viral glycoproteins in multiple functional states. HDX-MS, oxidative labeling, SAXS and EM will continue to provide unprecedented insight into the most dynamic stages of the infectious cycles of viruses as few other techniques are capable of probing these states in such detail. Additionally, approaches such as single-molecule fluorescence with structure-specific Förster resonance energy transfer labels, while low in specific structural information content offer tremendous insight into dynamics of conformational sampling and enable protein function to be directly observed as has been demonstrated for HIV Env on the surface virus particles [12,14]. In addition, synergy is gained between these and classical methods when information from multiple sources can be combined to generate a far richer, more complete picture of the structure and function of these dynamic, variable and complex viral glycoproteins than any single method alone can provide [210]. Integrative structural biology of viral glycoproteins is thus poised to move from the realm of providing static snapshots of beginning and end states towards shedding light on the dynamic processes and conformational changes that drive their function.
